# A Group of Highly Secretory miRNAs Correlates with Lymph Node Metastasis and Poor Prognosis in Oral Squamous Cell Carcinoma

**DOI:** 10.3390/biom14020224

**Published:** 2024-02-15

**Authors:** Yicun Li, Yuntao Lin, Xiaolian Li, Yuling Chen, Gang Chen, Hongyu Yang

**Affiliations:** 1 Department of Oral and Maxillofacial Surgery, Peking University Shenzhen Hospital, Shenzhen Peking University-The Hong Kong University of Science and Technology Medical Center, Shenzhen 518035, China; liyicun@whu.edu.cn (Y.L.);; 2 State Key Laboratory of Oral & Maxillofacial Reconstruction and Regeneration, Key Laboratory of Oral Biomedicine Ministry of Education, Hubei Key Laboratory of Stomatology, School and Hospital of Stomatology, Wuhan University, Wuhan 430079, China

**Keywords:** small extracellular vesicles, oral squamous cell carcinoma, microRNA, motif, prognosis

## Abstract

MicroRNAs (miRNAs) in oral squamous cell carcinoma (OSCC)-derived small extracellular vesicles (sEVs) play a pivotal role in modulating intercellular communications between tumor cells and other cells in the microenvironment, thereby influencing tumor progression and the efficacy of therapeutic interventions. However, a comprehensive inventory of these secretory miRNAs in sEVs and their biological and clinical implications remains elusive. This study aims to profile the miRNA content of OSCC cell line sEVs and computationally elucidate their biological and clinical relevance. We conducted miRNA sequencing to compare the miRNA profiles of OSCC cells and their corresponding sEVs. Our motif enrichment analysis identified specific sorting motifs that are implicated in either cellular retention or preferential sEV secretion. Target cell analysis suggested that the sEV miRNAs potentially interact with various immune cell types, including natural killer cells and dendritic cells. Additionally, we explored the clinical relevance of these miRNAs by correlating their expression levels with TNM stages and patient survival outcomes. Intriguingly, our findings revealed that a distinct sEV miRNA signature is associated with lymph node metastasis and poorer survival in patients in TCGA-HNSC dataset. Collectively, this research furthers our understanding of the miRNA sorting mechanisms in OSCC and underscores their clinical implications.

## 1. Introduction

Oral squamous cell carcinoma (OSCC) ranks among the most prevalent malignancies globally, accounting for a substantial number of new cases and fatalities annually [1]. OSCC is characterized by a variety of histological subtypes, including conventional squamous, basaloid squamous, spindle cell, verrucous, papillary squamous, adenoid squamous and adenosquamous cell carcinoma. Among these, oral adenosquamous cell carcinoma (OAsCC) is a highly metastatic and invasive subtype of oral squamous cell carcinoma [2]. Regrettably, a significant proportion of OSCC diagnoses occur at an advanced stage, correlating with a poor prognosis [3]. The overall 5-year survival rate for OSCC patients remains at a modest 50–60%, with the rate dropping to 10–40% for those with late-stage disease [4]. These statistics underscore the urgent need for innovative therapeutic strategies to enhance OSCC management. Advancements in OSCC treatment hinge on a comprehensive understanding of tumor cell biology, particularly the mechanisms by which these malignant cells manipulate their microenvironment to promote their own survival. Accumulating evidence has demonstrated that tumor-derived sEVs can alter their microenvironment and impact the immune response and metastasis. For example, OSCC cells have been shown to reprogram macrophages, transforming them into tumor-associated macrophages (TAMs) that actively contribute to tumor progression [5]. Moreover, OSCC cells can recruit and reprogram single-nucleus cells into natural killer cells (NK cells), which effectively dampen the anti-tumor immune responses [6]. Recent studies demonstrate that tumor-derived sEVs can dampen the effects of immunotherapy by suppressing CD8 T cells [7,8]. Other studies have demonstrated the critical role of tumor–immune interactions in cancer metastasis, highlighting the necessity to unravel the mechanisms through which tumor cells modulate immune cells via sEVs [9]. A pivotal role in the repurposing of other cells is played by tumor-derived small extracellular vesicles (sEVs) that traffic proteins, RNA, DNA, and lipids [10,11]. Bioactive molecules in tumor-derived sEVs mediate a complex network of cell-to-cell signaling pathways, with far-reaching implications for disease progression and potentially, therapeutic intervention. In fact, various studies have shown that tumor-derived sEV-mediated intercellular communication can exacerbate disease states and hamper therapeutic interventions [7,12,13,14,15]. Thus, inhibiting these tumor-derived sEV-mediated communications represents a promising frontier for alleviating the pathogenic interactions that fuel tumor progression [16,17]. However, our current knowledge of the microRNA (miRNA) repertoire in the OSCC secretome is incomplete, particularly regarding what is inside tumor-derived sEVs and what cell types might be their target cells. This incomplete knowledge hampers our efforts to utilize this mechanism for tumor treatment. Thus, further research is warranted to understand the relationships between OSCC cells and their surrounding cells. Considering the necessity to obtain a complete map of the “miRNA secretome” in an OSCC-specific context, we propose to construct an miRNA secretory profile and evaluate the miRNAs’ biological and clinical relevance, including their roles in intercellular communication.

In the current study, we extracted sEVs from two cultured OSCC cell lines, CAL27 and SCC25. We performed miRNA-seq of the cellular and sEV miRNAs to illustrate the miRNA profiling of the OSCC secretome. We identified a subset of highly secretory miRNAs by comparing their abundances between sEVs and cellular miRNAs. We analyzed the sequence basis of the secretory selectivity and evaluated the potential target cells and functions of the sEV secretory miRNAs. In addition, by applying our sEV secretory miRNA signature to the publicly available TCGA cohort, we evaluated the clinical relevance of these miRNAs. Our results provide insights into how OSCC cells regulate surrounding cells by selectively secreting miRNAs into sEVs.

## 2. Materials and Methods

### 2.1. Cell Culture

The human oral adenosquamous cell carcinoma cell line CAL27 and human oral squamous cell carcinoma cell line SCC25 were propagated under controlled conditions [18]. They were cultured in Dulbecco’s Modified Eagle Medium (DMEM) with a high glucose concentration and supplemented with 10% fetal bovine serum (FBS). To maintain optimal growth conditions, both cell lines were incubated in a humidified environment at 37 °C with an atmosphere containing 5% CO_2_.

### 2.2. Isolation of EVs from Cultured Cells

Extracellular vesicles (EVs) were harvested from cell culture supernatants using an ultracentrifugation protocol. First, we centrifuged the supernatants twice at 3000× *g* for 20 min at 4 °C to remove any cell debris. Then, the cleared supernatant underwent ultracentrifugation at 120,000× *g* for 70 min at 4 °C using a Beckman Coulter Optima XE-100 (Brea, CA, USA), facilitating EV isolation. The EV pellet was resuspended in phosphate-buffered saline (PBS). The quantitative and qualitative analysis of the EVs was conducted through nanoparticle tracking analysis (NTA, Particle Metrix, Ammersee, Germany). For transmission electron microscopy (TEM) examination, EV samples were applied to carbon-coated grids for 2 min, followed by two PBS washes. Post blotting and air-drying, the specimens were stained with 2% uranyl acetate for enhanced contrast and visualized using a Hitachi transmission electron microscope (Chiyoda City, Japan).

### 2.3. miRNA Sequencing and Analysis

Library construction of the miRNA sequencing was performed using a QIAseq miRNA library kit (Qiagen, Germantown, MD, USA) as per the manufacturer’s instructions. Three biological replicates were used for each group. Quality controls of the raw FASTQ files were included using the FastQC software (version 0.12). This step was followed by the trimming of adapters and low-quality bases using Trimmomatic (version 0.32) [19]. The processed reads were then mapped to the reference transcriptome using the gapped-aligner tool STAR (version 2.7.10b) [20] with the following parameters: outFilterMultimapNmax 10, outFilterMultimapScoreRange 0, outFilterScoreMinOverLread 0, outFilterMatchNmin 16, outFilterMatchNminOverLread 0, outFilterMismatchNmax 1, alignIntronMax 1, alignSJDBoverhangMin 1000, alignEndsType EndToEnd, and sjdbGTFfile microRNA.subset.of.GENCODE.V24.gtf. Sequencing depths were verified to ensure alignment with the established standards [21]. Post mapping, the aligned reads were assembled and quantified with the aid of StringTie (version 1.9) [22]. The differential expression analysis of the genes was executed using the DESeq2 software package (version 1.34) [23]. Initially, we imported the miRNA count matrix through tximport (version 3.18) and constructed the DESeqDataSe object [24]. The DESeq function was then applied to normalize the miRNA count data. Subsequently, the results of the differential expression analysis were obtained using the results function. miRNA location enrichment analysis was performed using the miEAA (version 2.0) platform with the RNALocate (version 2.0) database [25,26].

### 2.4. Phylogenetic Analysis

We focused on mature microRNAs that were significantly enriched (adjusted *p* value < 0.05) in cells (sEVs vs. cell, log_2_fold change < −1) or in sEVs (sEVs vs. cell, log_2_fold change > 1) for the phylogenetic analysis. First, we used CLUSTAL W (version 2.1) for multiple sequence alignments [27]. Then, we used a RAxML bootstrap to construct the phylogenic trees. Visualization of the phylogenic tree was performed with FigTree (v1.4.4) [28].

### 2.5. Motif Enrichment Analysis Using the Gene Set Enrichment Analysis (GSEA) Algorithm

For motif enrichment analysis, we created gene set files that encapsulated all potential permutations of nucleotide sequences (motifs) ranging in length from 4 to 7 bases. Motifs containing fewer than 6 genes are filtered out. Then, we established a ranked list of genes, ordered according to their log_2_fold change. This ranking was generated using DESeq2, facilitating a comparative analysis of the relative abundance of miRNAs in small extracellular vesicles (sEVs) versus their cellular counterparts. We then performed a pre-ranked GSEA analysis to identify specific motifs that demonstrated a pronounced tendency for either secretion into sEVs or retention within the cells [29].

### 2.6. RNA-Binding Protein Enrichment Analysis

For the enrichment analysis of RNA-binding proteins (RBPs), we utilized miRNA–RBP interaction data from the RNAInter database (version 4.0, http://www.rnainter.org/, accessed on 10 May 2023) [30]. We determined the frequency of RBPs interacting with either background or compartment-enriched miRNAs. To assess the significance of the observed versus expected frequency of these miRNA interactions, we applied the chi-squared test. Subsequently, we adjusted the *p* values to account for multiple tests. Finally, we identified the RBPs that were over-represented in sEVs or cellular compartments.

### 2.7. sEV miRNA Target Cell Type Prediction Analysis

For predicting target cell types of OSCC sEV miRNAs, we first acquired miRNA target mRNA information from the RNAInter database (version 4.0, http://www.rnainter.org/, accessed on 10 May 2023). Then, we constructed an miRNA–target mRNA network using Cytoscape (version 3.10.1) [31]. Subsequently, we acquired cell type-specific mRNAs from the CellMarker database (version 2.0, http://117.50.127.228/CellMarker/, accessed on 31 May 2023) [32]. Then, we employed a hypergeometric test to identify significant cell types associated with miRNA target mRNAs.

### 2.8. sEV miRNA Functional Enrichment Analysis

For functional enrichment analysis of OSCC sEV miRNAs, we used the miEAA (version 2.0) platform with the Kyoto Encyclopedia of Genes and Genomes (KEGG, version 2.0) database and Molecular Signatures Database (MSigDB) Hallmark datasets [29,33].

### 2.9. TCGA miRNA Expression Analysis

First, we downloaded the TCGA-HNSC miRNA expression and clinical data from the Genomic Data Commons Data Portal (GDC, https://portal.gdc.cancer.gov/, accessed on 5 July 2023) [34]. A *t*-test was used to compare the expression difference between the normal and tumor samples. A Kaplan–Meier curve and log-rank test were used in a survival analysis between the high- and low-expression subgroups. The cut-off point chosen for the survival analysis was the one yielding the lowest *p* values. For the analysis of miRNA expression across the TNM stages, we utilized the pathological T and N stages.

### 2.10. Statistical Analysis

The variances among the multiple groups were analyzed using a one-way ANOVA and post hoc Tukey test. An unpaired *t*-test was employed to evaluate any differences between the two groups. *p* < 0.05 was considered statistically significant. All statistical analyses were performed using GraphPad Prism software (version 10.1.1, Graph Pad Software Inc., La Jolla, CA, USA).

## 3. Results

### 3.1. Profiling the miRNA Secretome in OSCC sEVs

**Figure 1:**
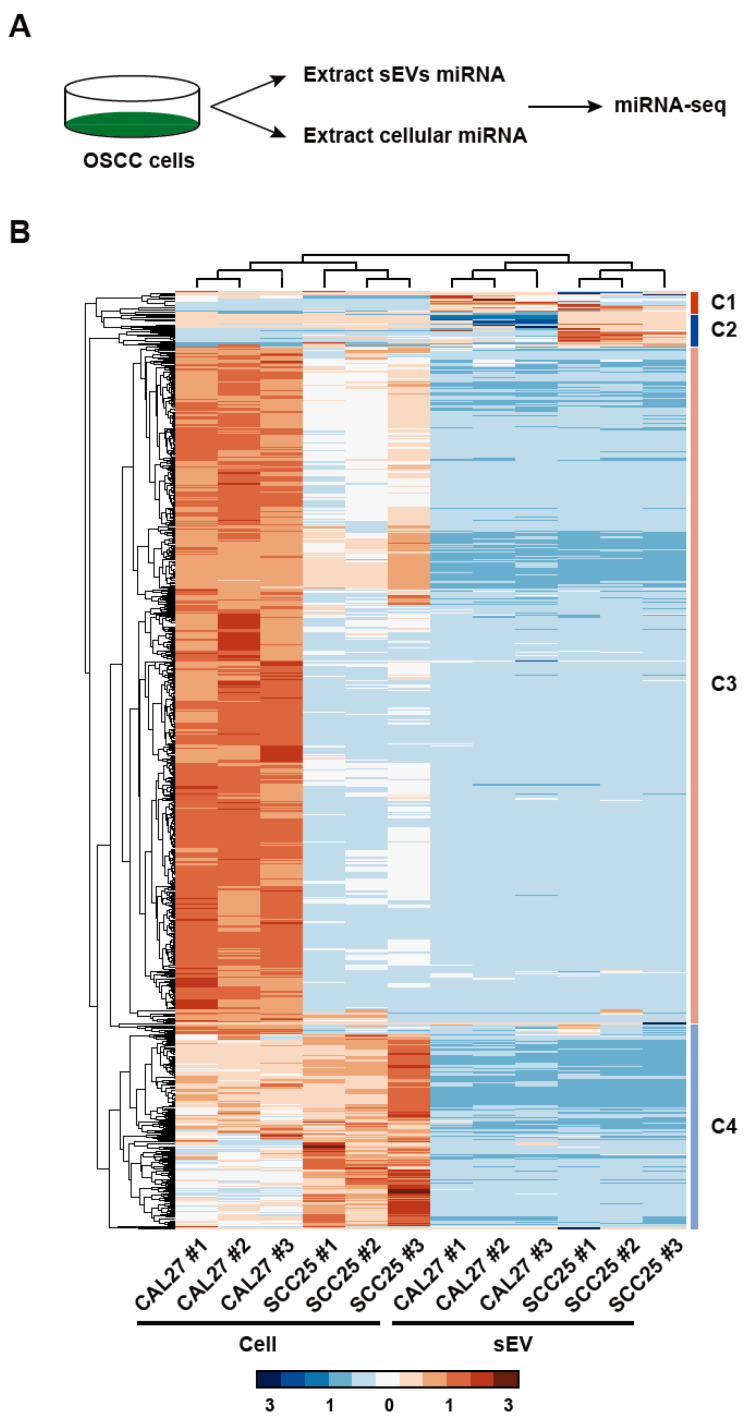
Analysis of the miRNA profiles in OSCC cells and sEVs. (**A**) Schematic representation of the experimental workflow. miRNA was extracted from OSCC cells and sEVs, followed by miRNA sequencing to compare the miRNA profiles between the cells and sEVs. (**B**) Heatmap illustrating the differential miRNA expression profiles in OSCC cell lines (CAL27 and SCC25) and their corresponding sEVs. Each row represents a unique miRNA, and each column represents a sample from either the cellular miRNA or sEV miRNA. The scale bar at the bottom indicates the expression level of the miRNAs. The dendrogram on the left clusters miRNAs into four main groups (C1 to C4). The dendrogram on the top clusters the samples according to their miRNA expression similarity.

First, we isolated sEVs from two OSCC cell lines, CAL27 and SCC25, and then we characterized the sEVs using nanoparticle tracking analysis and transmission electron microscopy (Supplementary Materials Figure S1A). Then, we extracted total RNA from whole cells and sEVs and performed miRNA-seq (Figure 1A). After data preprocessing and abundance calculations, we identified a total of 2918 miRNAs. A total of 1680 miRNAs were detected within sEVs, with 62.98% (1058 miRNAs) being common to sEVs from both OSCC cell lines. In cellular miRNA profiling, a total of 2887 miRNAs were identified, with 66.81% (1528 miRNAs) shared between the two cell lines. Notably, a high percentage of miRNAs present in the CAL27 sEVs (95.42%, 1333/1397) and SCC25 sEVs (92.24%, 1237/1341) were also detectable in their respective cellular counterparts, as further detailed in Supplementary Figure S1B,C. Quantitative analysis of the miRNA secretome revealed distinct clustering patterns in the miRNA expression levels between cells and sEVs, as shown in Figure 1B. This unsupervised clustering resulted in the identification of four major miRNA clusters (C1–C4), each corresponding to sEVs with high enrichment in either of the two cell lines or sEVs. The miRNAs most significantly enriched in the sEVs included hsa-mir-24-1, hsa-miR-103b, and hsa-miR-127, while those most significantly retained within cells were hsa-mir-181a, hsa-miR-7974, and hsa-mir-24-2 (Supplementary Table S1). Cellular compartment enrichment analysis against the RNAlocate database showed that the C1 and C2 clusters are mainly located in microvesicles and circulating compartments (Supplementary Figure S1D). Additionally, ranking genes based on the ratio of their expression levels between sEVs and cells highlighted miRNAs with either a propensity for high secretion or retention within cells (Supplementary Figure S1E). The choice of destination for the miRNAs seems not to be randomly chosen, with the phylogenetic analysis showing that the highly secretory miRNAs and miRNAs retained in cells clustered together (Supplementary Figure S1F). These findings underscore the complexity of the miRNA distribution in OSCC, indicating that while a substantial proportion of cellular miRNAs are able to be secreted into sEVs, only a select few are significantly enriched within these vesicles.

### 3.2. RNA Motifs for miRNA Secretion or Retention

**Figure 2:**
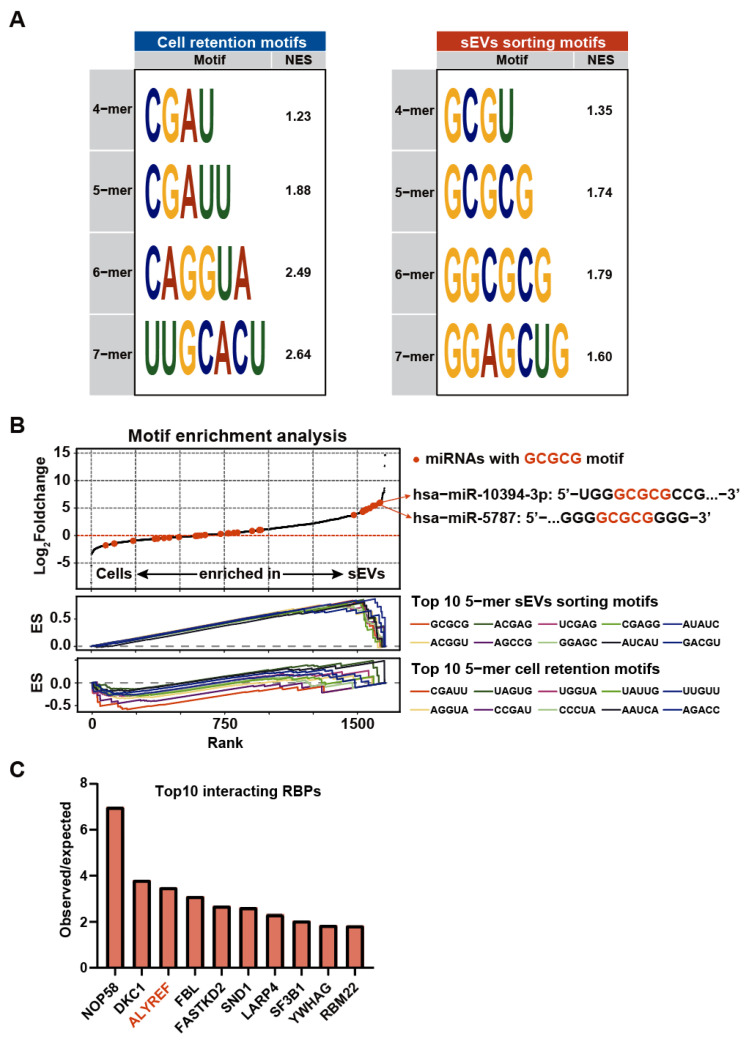
Motif enrichment analysis of secretory and cellular miRNAs. (**A**) Motifs enriched in miRNAs retained in OSCC cells or sorted into sEVs. The left panel shows 4-mer to 7-mer cell retention motifs. The right panel shows sEV 4-mer to 7-mer sEV-sorting motifs. The normalized enrichment score (NES) is provided for each motif, indicating the degree of enrichment for each motif. (**B**) The top graph shows the log_2_fold change in miRNA expression. The red dots represent miRNAs containing the GCGCG motif. The middle and bottom graphs show the running enrichment scores of the top 10 5-mer sEV-sorting and cell retention motifs, respectively. Each motif is represented by a different colored line, illustrating the motif distribution across the ranked miRNAs. (**C**) The top 10 enriched RBPs of the secretory miRNAs.

Recent studies have shown that miRNA secretion into sEVs is governed by certain RNA motifs and RNA-binding proteins [35,36]. In order to identify key motifs associated with miRNA secretion into sEVs or retention within cells, we performed a motif enrichment analysis using GSEA. We compared the motifs of miRNAs present in sEVs with those of cellular miRNAs. As shown in Figure 2A, the core motif “GCGC” is predominantly associated with miRNA secretion into sEVs. The core motif “CGAU” is predominantly associated with miRNA retention in cells. miRNAs containing these core motifs (such as hsa-miR-10394-3p and has-miR-5787) were found to be enriched either in cells or sEVs (Figure 2B). Further analysis of cell-line-specific motifs showed that each cell line possesses their own sEV-sorting and cell retention motifs (Supplementary Figure S2A,B). The diversity of sEV-sorting sequences may be due to the available RNA-binding proteins (RBPs) that might mediate miRNA sorting into sEVs. In order to identify possible RBPs responsible for miRNA sEV sorting in OSCC cell lines, we conducted miRNA–RBP interaction enrichment analysis on miRNAs enriched in either cells or sEVs. The top 10 most enriched (observed vs. expected) RPBs are shown in Figure 2C. Some RBPs have been established as miRNA-sorting proteins (such as ALYREF1) [36]. We also found previously unreported RBPs (such as NOP58, DKC1, etc.). These results indicate that miRNA sorting in OSCC cells may be regulated by RBPs and the corresponding miRNA motifs.

### 3.3. Functional Annotation of the sEV miRNAs

**Figure 3:**
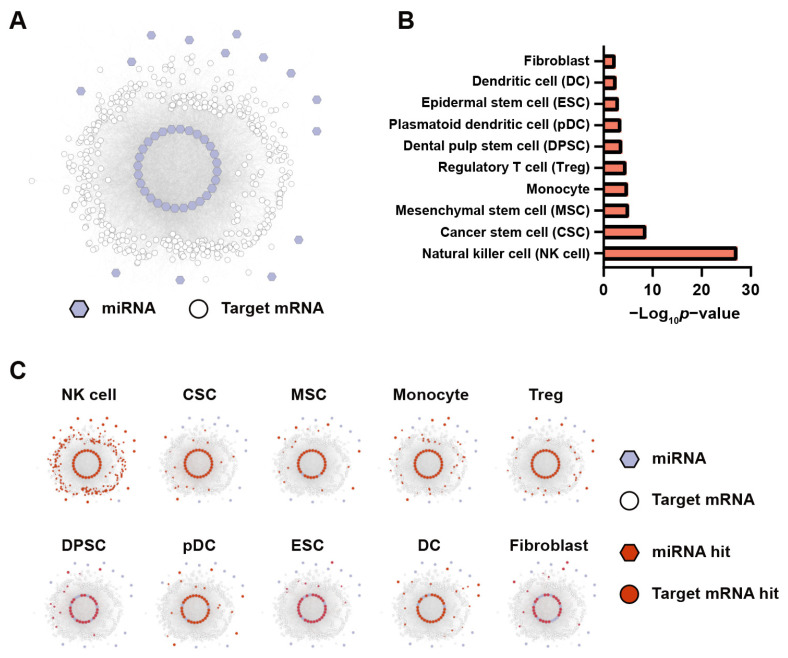
Analysis of sEV-enriched miRNA target mRNAs and their cell type enrichment. (**A**) A network plot representing sEV-enriched miRNAs (blue hexagons) and their target mRNAs (white circles). (**B**) A bar graph showing the cell type enrichment analysis. −Log_10_*p* value indicates the significance of the association between the target mRNAs and the specific cell types based on the CellMarker database. (**C**) The network plot displays the detailed miRNAs and their target mRNAs involved in each cell type, as denoted in red. Each subplot corresponds to a different cell type.

In order to discover the functional impact of these secretory miRNAs, it is helpful to know the target cell types of the sEV miRNA. We hypothesized that miRNAs targeting mRNAs is enriched in certain cell types. By analyzing the cell types of the enriched mRNA targets, we can infer what cell types those sEVs are targeting. We classified miRNAs significantly enriched in sEVs (adjusted *p* value < 0.05 and log_2_fold change >0) as sEV secretory miRNAs. Additionally, miRNAs significantly enriched in cells (adjusted *p* value < 0.05 and log_2_fold change < 0) were classified as cell retention miRNAs. First, we employed a network analysis of sEV-enriched miRNAs and their target mRNAs (Figure 3A). Then, we performed a hypergeometric analysis of the target mRNAs in the CellMarker database (Supplementary Table S2). The results showed that most of the enriched cell types included several types of immune cells (such as NK cells, regulatory T cells and dendritic cells), cancer stem cells, mesenchymal cells and dental pulp stem cells. Among them, the most significant cell type was NK cells (Figure 3B). The detailed analysis showed that most miRNAs enriched in OSCC sEVs target mRNAs abundant in NK cells (Figure 3C). The KEGG pathway enrichment analysis showed that these target mRNAs were mainly targets of miRNAs abnormally expressed by cancer cells (Supplementary Figure S3A). The molecular function enrichment analysis showed that the most significant functions of these target mRNAs were TGF-β signaling, apoptosis and adipogenesis (Supplementary Table S3). These results indicate that NK cells and other immune cells may be the primary targets of OSCC sEVs.

### 3.4. Clinical Relevance of the Identified OSCC sEV miRNA Signatures

**Figure 4:**
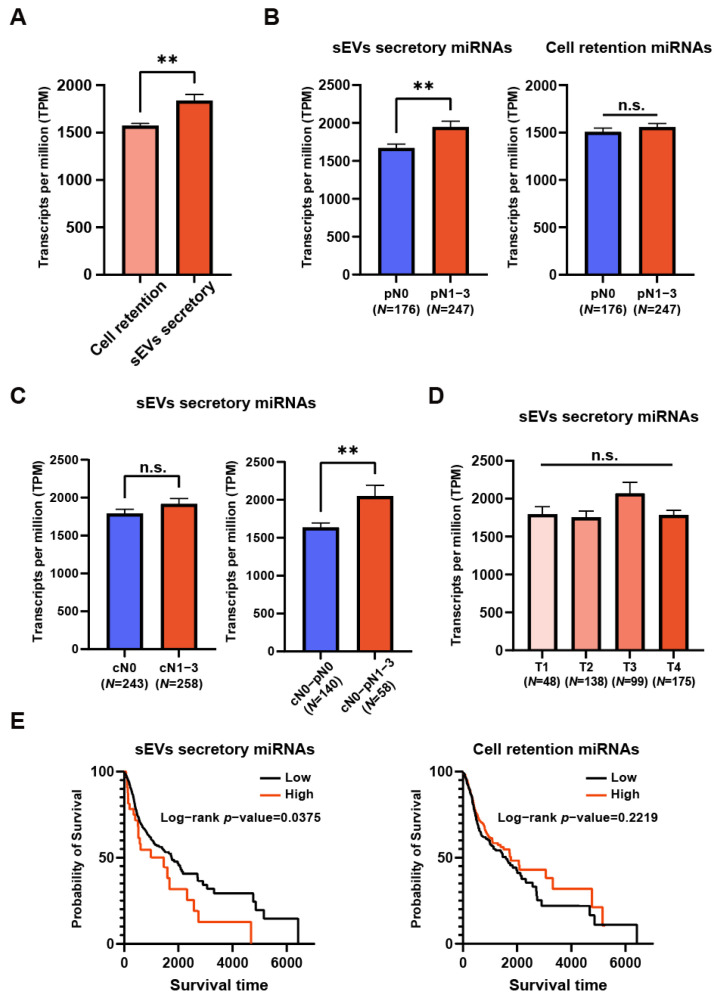
The clinical relevance of sEV secretory and cellular retention miRNAs in the TCGA-HNSC dataset. (**A**) Comparison of the expression levels revealed that sEV secretory miRNAs were significantly more highly expressed in tumor samples compared to cell retention miRNAs. ** *p* value < 0.01. (**B**) The expression levels of sEV secretory and cell retention miRNAs in patients with (N0) or without (N1-3) lymph node metastasis. ** *p* value < 0.01. n.s. not significant. (**C**) The expression levels of sEV secretory miRNAs across patient groups. ** *p* value < 0.01. n.s. not significant. (**D**) The expression levels of sEV secretory and cell retention miRNAs in patients with different tumor stages (T1-T4). n.s. not significant. (**E**) The Kaplan–Meier survival plots of sEV secretory and cell retention miRNAs, grouped by low and high expression levels.

To assess the clinical relevance of the OSCC sEV secretory miRNA signatures, we analyzed publicly available miRNA expression data from the TCGA-HNSC dataset, which are predominantly composed of data from oral squamous cell carcinoma. Our analysis revealed that sEV secretory miRNAs are more abundant in tumor tissues compared to cell retention miRNAs (Figure 4A). Interestingly, paired normal mucosa demonstrated a higher expression of sEV secretory miRNAs and a reduced expression of cell retention miRNAs (Supplementary Figure S4A). A notable association was observed between the elevated levels of sEV secretory miRNAs and advanced pathological N stages, with the highest expression noted at the pN2 stage. This correlation was not evident with cell retention miRNAs (Figure 4B and Figure S4B). Further analysis showed that although no significant differential expression of sEV secretory miRNAs was observed between clinical N0 and N1-3 stages, their expression was significantly higher in clinically non-metastatic but pathologically metastatic (occult metastasis) patients (Figure 4C). Furthermore, our study found no correlation between the expression of sEV secretory or cell retention miRNAs with the pathological T stages of OSCC (Figure 4D and Figure S4C). Additionally, the sEV secretory miRNA signature was significantly correlated with a poorer prognosis in patients. While the cell retention miRNA signature was correlated with a better prognosis in patients, the result was not statistically significant (Figure 4E). We validated our sEV secretory miRNA signature in additional OSCC datasets, GSE216630 (Supplementary Figure S4D) [37]. Collectively, these findings indicate a positive correlation of secretory miRNAs with lymph node metastasis and a poorer prognosis. Secretory miRNA expression profiles also have potential use in distinguishing between occult metastatic and non-metastatic disease states.

## 4. Discussion

Profiling miRNA secreted by tumor cells into small extracellular vesicles and those retained within cells is crucial for understanding the intercellular communication between tumor cells and the surrounding microenvironment. Recent research has demonstrated that a selective subset of miRNAs expressed in cells is preferentially enriched in sEVs [36]. Additionally, our profiling has shown a similar pattern with other studies regarding miRNAs in tumor-derived small extracellular vesicles [37,38]. This selective secretion process involves specific RNA-binding proteins that facilitate miRNA incorporation into sEVs [35,36]. The concept of ‘sEV secretory miRNAs’ arises from these observations, though the mechanisms governing miRNA sorting into sEVs remain incompletely understood. Possible mechanisms include nSMase2-, RBP-, poly(U)- or miRISC-dependent pathways [39]. Emerging evidence suggests that a core GGAG motif plays a pivotal role in miRNA sorting into sEVs. Engineering an miRNA with this nucleic acid motif can enhance its incorporation into sEVs [36,39,40]. Our research further corroborates the significance of the GGAG motif, identifying it as the most prominent 4-mer motif in CAL27 sEV-sorting motifs. Additionally, GGAG forms part of the 6-mer CAL27 sorting motif CGGAGC and the 7-mer motif GGAGCUG, common to both CAL27 and SCC25 sEVs. The top 10 5-mer common sorting motifs, including GGAGC, also feature this core GGAG sequence. The specific role of these motifs in miRNA sorting into sEVs is still under investigation, but existing evidence points towards a link to RNA-binding proteins. Known RBPs implicated in this process include hnRNPA2B1, Lupus La, Ybx1, and Alyref [35,36,41]. Our analysis identified Alyref as one of the enriched RBPs, but not other reported RBPs. We also identified LARP4, a La-related protein (LaRP) like Lupus La (also known as LARP3), suggesting novel participants in miRNA sorting into sEVs. 

Research has established that small extracellular vesicles (sEVs) are not uniformly distributed within the circulatory system or the microenvironment, exhibiting a preference for specific target cells [42,43]. sEVs are composed of a diverse array of proteins, lipids, and RNAs. One important side of the functionality of sEVs is attributed to their surface proteins. In contrast, miRNAs within sEVs become functional only upon uptake by recipient cells. While the target cells of sEV surface proteins can be inferred based on their interactions with receptors or ligands (e.g., PD-L1 to PD-1), predicting the target cells for miRNAs is more challenging. This aspect of sEV-mediated communication remains largely underexplored. To address this gap, our study employed a computational approach to predict the target cells of miRNAs based on the cell-type specificity of their target mRNAs. This method offers a novel approach for deducing potential target cells from the miRNA content of sEVs. However, these findings are preliminary and theoretical, and further extensive experimental validation is needed to confirm their accuracy and biological relevance.

Tumor cells tend to secrete more sEVs than normal cells [44]. This increased secretion has been shown to promote various malignant behaviors in tumors. The miRNAs within these sEVs are encapsulated in bi-layered lipid structures, providing them with resistance to the abundant nucleases present in the circulatory system and thus a prolonged half-life [45]. Studies have indicated that tumor-derived sEVs can navigate through lymphatic vessels and become internalized by diverse cell types within lymph nodes [46]. This could lead to the formation of pre-metastatic niches, potentially facilitating tumor metastasis [46,47]. Indeed, we found a unique OSCC sEV secretory miRNA signature that positively correlated with lymph node metastasis. Interestingly, we observed that sEV secretory miRNA expression was not significantly upregulated in clinically diagnosed lymph node metastasis, but it was markedly elevated in cases of pathologically confirmed metastasis. This suggests that the expression levels of sEV secretory miRNAs could serve as a discriminative marker, differentiating actual lymph node metastasis from false-positive clinical diagnoses. Furthermore, increased sEV secretory miRNA expression in patients clinically classified as node-negative (cN0) may indicate the presence of occult lymph node metastasis, underscoring the potential of these biomarkers in cancer diagnostics and prognostics. Mechanistically, EMT plays a pivotal role in the progression of cancer metastasis, allowing epithelial tumor cells to behave like mesenchymal cells, thereby enhancing their mobility and invasive capabilities. miRNAs in tumor-derived sEVs are known to trigger EMT in OSCC cells through the inhibition of epithelial marker genes and the induction of mesenchymal marker genes [48]. Furthermore, sEV miRNAs could induce angiogenesis, thereby promoting the development of new blood vessels that support tumor expansion and the spread of tumor cells [49]. sEVs also play a crucial role in establishing pre-metastatic niches at distant sites by modifying the local microenvironment to favor the settlement of metastasizing tumor cells [50]. Moreover, miRNAs associated with sEVs can modulate the immune landscape within the tumor microenvironment, facilitating OSCC cells to evade immune surveillance. This is achieved by specific miRNAs that can inhibit the activity of immune cells, such as T cells and natural killer cells, or drive macrophages towards a phenotype that supports tumor growth [51,52,53].

This study has certain limitations that need to be considered. Firstly, the results are derived from only two OSCC cell lines, which may not fully represent the heterogeneity of OSCC. The histological types of these cell lines are limited to adenosquamous and squamous cell carcinoma, which do not represent the full range of histological variations in OSCC. Our in vitro monolayer culture method is not able to replicate the intricate, multi-cellular tumor microenvironment. Additionally, while the findings offer valuable insights into miRNA dynamics in OSCC, the exact biological implications and mechanisms underlying these observations need functional validation in animal models and larger patient cohorts, particularly their contribution to tumor progression, metastasis and prognosis. Moreover, while we discovered several motifs associated with miRNA sorting into sEVs, experimental validation involving site mutation of the motifs needs to be conducted. The use of small extracellular microRNAs as biomarkers is challenged by issues such as specificity, sensitivity, lack of standardized protocols, and biological variability, all of which complicate their detection, quantification, and interpretation in disease diagnosis and monitoring [54]. Another limitation is that our in silico predictions of the target cell types for sEV miRNAs might not accurately reflect real-life in vivo conditions. Therefore, experimental validation is necessary to confirm the predicted target cells.

The emerging field of small extracellular vesicle (sEV) research has significantly advanced our understanding of tumor biology and intercellular communication. The selective secretion of miRNAs into sEVs by tumor cells, as well as their specific targeting to certain cells, highlights a complex mechanism of intercellular signaling that plays a critical role in cancer progression. The discovery of specific motifs that influence sEV sorting underscores the nuanced nature of these processes. Biological studies, together with clinical research, will lead to improved diagnostic and prognostic tools in oncology, enhancing our ability to effectively detect and treat cancer.

## 5. Conclusions

In this investigation, we employed miRNA sequencing to analyze both cellular and small extracellular vesicle miRNAs derived from OSCC cell lines. We employed a series of analyses to delineate miRNA expression profiles and discover the association between sEV-derived miRNA expression levels and various biological and clinicopathological features. We identified a distinct, highly selective subset of miRNAs in OSCC cell lines. Moreover, the motif analysis suggested that this selectivity could be attributed to the motif-binding activities of RNA-binding proteins. Network and target cell prediction analyses revealed that sEV-derived miRNAs in OSCC predominantly target immune cells, including natural killer cells, regulatory T cells, and dendritic cells. Additionally, analysis of miRNA expression data from the TCGA-HNSC dataset demonstrated that the signature of the OSCC-derived sEV miRNAs was significantly associated with lymph node metastasis and an adverse prognosis.

## Data Availability

The miRNA-seq data presented in this study were deposited in the Gene Expression Omnibus (GEO) repository (accession number: GSE254937).

## Supplementary Materials

The following supporting information can be downloaded at https://www.mdpi.com/article/10.3390/biom14020224/s1, Figure S1: Analysis of miRNA profiles in OSCC cells and sEVs. (**A**) TEM images and NTA analysis of sEVs extracted from CAL27 and SCC25. Scale bar represents 100 nanometers. (**B**) Venn diagram showing the overlap and specificity of miRNAs between CAL27 cells, CAL27-derived sEVs, SCC25 cells, and SCC25-derived sEVs. The total number of identified miRNAs is shown. (**C**) Pie charts comparing the percentage of shared and cell line-specific miRNAs in cells and sEVs. The left pie chart represents the distribution of miRNAs in sEVs. The right pie chart represents the distribution of miRNAs in cells. (**D**) UpSet plot shows the RNA location enrichment analysis of C1 and C2 group miRNAs. (**E**) Ranked plot showing the fold-change of miRNA abundance in sEVs versus cells. The x-axis represents the rank of miRNAs based on differential abundance; the y-axis shows the log-transformed fold-change (sEVs vs cells). The highlighted yellow area indicates miRNAs with significant differential expression. (**F**) Phylogenetic tree displaying the clustering of miRNAs based on their sequence homology. sEVs miRNAs were colored in red and cellular miRNAs were colored in blue. Orange block denotes sEVs miRNAs dominant clusters and blue block denotes cellular miRNA dominant clusters. Motif enrichment analysis of secretory and cellular miRNAs specific to CAL27 and SCC25. Figure S2: (**A**) Motifs enriched in miRNAs retained in OSCC cells or sorted into sEVs, specific to CAL27 and SCC25, respectively. Normalized enrichment score (NES) is provided for each motif, indicating the degree of enrichment of each motif. (**B**) Running enrichment score plot of top 10 5-mer sEVs sorting and cell retention motifs, respectively. Red dots represent miRNAs containing a specific motif. Each colored line illustrates one of the top motif distributions across the ranked miRNAs. Figure S3: Functional enrichment analysis of sEVs miRNAs. (**A**) KEGG pathway enrichment analysis of sEVs miRNAs. (**B**) Molecular function enrichment (MSigDB Hallmark) analysis of sEVs miRNAs. Bars represented −log_10_*P*-value, indicating the significance of each pathway’s enrichment. Lines showed the corresponding odds ratio for each pathway. Figure S4: Clinical relevance of sEVs secretory and cellular retention miRNAs in TCGA-HNSC dataset. (**A**) Expression levels of sEVs secretory and cell retention miRNAs in normal and tumor tissues. **** *p*-value < 0.0001. (**B**) Expression levels of sEVs secretory and cell retention miRNAs in patients with different N stages (N0-N3). (**C**) Expression levels of sEVs secretory and cell retention miRNAs in patients with different T stages (T1–T3). (**D**) Expression levels of sEVs secretory and cell retention miRNAs in patients with different N stages (N0 or N+) in GSE216630 dataset. * *p*-value < 0.05. n.s. not significant. Table S1: Differentially enriched sEVs in sEVs and cells. Table S2: Cell type enrichment analysis. Table S3: Functional enrichment analysis.

## References

[1] Tan Y., Wang Z., Xu M., Li B., Huang Z., Qin S., Nice E.C., Tang J., Huang C. (2023). Oral squamous cell carcinomas: State of the field and emerging directions. Int. J. Oral Sci..

[2] Sravya T., Rao G.V., Kumar M.P., Sudheerkanth K. (2016). Oral adenosquamous carcinoma: Report of a rare entity with a special insight on its histochemistry. J. Oral Maxillofac. Pathol..

[3] Mauceri R., Bazzano M., Coppini M., Tozzo P., Panzarella V., Campisi G. (2022). Diagnostic delay of oral squamous cell carcinoma and the fear of diagnosis: A scoping review. Front. Psychol..

[4] Tsai Y.-T., Chen W.-C., Hsu C.-M., Tsai M.-S., Chang G.-H., Lee Y.-C., Huang E.I., Fang C.-C., Lai C.-H. (2021). Survival-Weighted Health Profiles in Patients Treated for Advanced Oral Cavity Squamous Cell Carcinoma. Front. Oncol..

[5] Cai J., Qiao B., Gao N., Lin N., He W. (2019). Oral squamous cell carcinoma-derived exosomes promote M2 subtype macrophage polarization mediated by exosome-enclosed miR-29a-3p. Am. J. Physiol.-Cell Physiol..

[6] Zhu X., Qin X., Wang X., Wang Y., Cao W., Zhang J., Chen W. (2020). Oral cancer cell-derived exosomes modulate natural killer cell activity by regulating the receptors on these cells. Int. J. Mol. Med..

[7] Chen G., Huang A.C., Zhang W., Zhang G., Wu M., Xu W., Yu Z., Yang J., Wang B., Sun H. (2018). Exosomal PD-L1 contributes to immunosuppression and is associated with anti-PD-1 response. Nature.

[8] Kim D.H., Kim H., Choi Y.J., Kim S.Y., Lee J.-E., Sung K.J., Sung Y.H., Pack C.-G., Jung M.-k., Han B. (2019). Exosomal PD-L1 promotes tumor growth through immune escape in non-small cell lung cancer. Exp. Mol. Med..

[9] Garner H., de Visser K.E. (2020). Immune crosstalk in cancer progression and metastatic spread: A complex conversation. Nat. Rev. Immunol..

[10] Qian K., Fu W., Li T., Zhao J., Lei C., Hu S. (2022). The roles of small extracellular vesicles in cancer and immune regulation and translational potential in cancer therapy. J. Exp. Clin. Cancer Res..

[11] Valcz G., Újvári B., Buzás E.I., Krenács T., Spisák S., Kittel Á., Tulassay Z., Igaz P., Takács I., Molnár B. (2022). Small extracellular vesicle DNA-mediated horizontal gene transfer as a driving force for tumor evolution: Facts and riddles. Front. Oncol..

[12] Fuentes P., Sesé M., Guijarro P.J., Emperador M., Sánchez-Redondo S., Peinado H., Hümmer S., Ramón y Cajal S. (2020). ITGB3-mediated uptake of small extracellular vesicles facilitates intercellular communication in breast cancer cells. Nat. Commun..

[13] Ye L., Zhu Z., Chen X., Zhang H., Huang J., Gu S., Zhao X. (2021). The Importance of Exosomal PD-L1 in Cancer Progression and Its Potential as a Therapeutic Target. Cells.

[14] Liu X., Wills C.A., Chen L., Zhang J., Zhao Y., Zhou M., Sundstrom J.M., Schell T., Spiegelman V.S., Young M.M. (2022). Small extracellular vesicles induce resistance to anti-GD2 immunotherapy unveiling tipifarnib as an adjunct to neuroblastoma immunotherapy. J. ImmunoTherapy Cancer.

[15] Zhong W., Xiao Z., Qin Z., Yang J., Wen Y., Yu Z., Li Y., Sheppard N.C., Fuchs S.Y., Xu X. (2023). Tumor-Derived Small Extracellular Vesicles Inhibit the Efficacy of CAR T Cells against Solid Tumors. Cancer Res..

[16] Dudiki T., Veleeparambil M., Zhevlakova I., Biswas S., Klein E.A., Ford P., Podrez E.A., Byzova T.V. (2023). Mechanism of Tumor-Platelet Communications in Cancer. Circ. Res..

[17] Altei W.F., Pachane B.C., Dos Santos P.K., Ribeiro L.N.M., Sung B.H., Weaver A.M., Selistre-de-Araújo H.S. (2020). Inhibition of αvβ3 integrin impairs adhesion and uptake of tumor-derived small extracellular vesicles. Cell Commun. Signal..

[18] Jiang L., Ji N., Zhou Y., Li J., Liu X., Wang Z., Chen Q., Zeng X. (2009). CAL 27 is an oral adenosquamous carcinoma cell line. Oral Oncol..

[19] Bolger A.M., Lohse M., Usadel B. (2014). Trimmomatic: A flexible trimmer for Illumina sequence data. Bioinformatics.

[20] Dobin A., Davis C.A., Schlesinger F., Drenkow J., Zaleski C., Jha S., Batut P., Chaisson M., Gingeras T.R. (2013). STAR: Ultrafast universal RNA-seq aligner. Bioinformatics.

[21] Jardillier R., Koca D., Chatelain F., Guyon L. (2022). Optimal microRNA Sequencing Depth to Predict Cancer Patient Survival with Random Forest and Cox Models. Genes.

[22] Pertea M., Pertea G.M., Antonescu C.M., Chang T.C., Mendell J.T., Salzberg S.L. (2015). StringTie enables improved reconstruction of a transcriptome from RNA-seq reads. Nat. Biotechnol..

[23] Love M.I., Huber W., Anders S. (2014). Moderated estimation of fold change and dispersion for RNA-seq data with DESeq2. Genome Biol..

[24] Soneson C., Love M., Robinson M. (2015). Differential analyses for RNA-seq: Transcript-level estimates improve gene-level inferences [version 1; peer review: 2 approved]. F1000Research.

[25] Cui T., Dou Y., Tan P., Ni Z., Liu T., Wang D., Huang Y., Cai K., Zhao X., Xu D. (2021). RNALocate v2.0: An updated resource for RNA subcellular localization with increased coverage and annotation. Nucleic Acids Res..

[26] Kern F., Fehlmann T., Solomon J., Schwed L., Grammes N., Backes C., Van Keuren-Jensen K., Craig D.W., Meese E., Keller A. (2020). miEAA 2.0: Integrating multi-species microRNA enrichment analysis and workflow management systems. Nucleic Acids Res..

[27] Thompson J.D., Higgins D.G., Gibson T.J. (1994). CLUSTAL W: Improving the sensitivity of progressive multiple sequence alignment through sequence weighting, position-specific gap penalties and weight matrix choice. Nucleic Acids Res..

[28] Stamatakis A. (2014). RAxML version 8: A tool for phylogenetic analysis and post-analysis of large phylogenies. Bioinformatics.

[29] Subramanian A., Tamayo P., Mootha V.K., Mukherjee S., Ebert B.L., Gillette M.A., Paulovich A., Pomeroy S.L., Golub T.R., Lander E.S. (2005). Gene set enrichment analysis: A knowledge-based approach for interpreting genome-wide expression profiles. Proc. Natl. Acad. Sci. USA.

[30] Kang J., Tang Q., He J., Li L., Yang N., Yu S., Wang M., Zhang Y., Lin J., Cui T. (2021). RNAInter v4.0: RNA interactome repository with redefined confidence scoring system and improved accessibility. Nucleic Acids Res..

[31] Shannon P., Markiel A., Ozier O., Baliga N.S., Wang J.T., Ramage D., Amin N., Schwikowski B., Ideker T. (2003). Cytoscape: A software environment for integrated models of biomolecular interaction networks. Genome Res..

[32] Zhang X., Lan Y., Xu J., Quan F., Zhao E., Deng C., Luo T., Xu L., Liao G., Yan M. (2018). CellMarker: A manually curated resource of cell markers in human and mouse. Nucleic Acids Research.

[33] Kanehisa M., Goto S. (2000). KEGG: Kyoto encyclopedia of genes and genomes. Nucleic Acids Res..

[34] Grossman R.L., Heath A.P., Ferretti V., Varmus H.E., Lowy D.R., Kibbe W.A., Staudt L.M. (2016). Toward a Shared Vision for Cancer Genomic Data. N. Engl. J. Med..

[35] Villarroya-Beltri C., Gutiérrez-Vázquez C., Sánchez-Cabo F., Pérez-Hernández D., Vázquez J., Martin-Cofreces N., Martinez-Herrera D.J., Pascual-Montano A., Mittelbrunn M., Sánchez-Madrid F. (2013). Sumoylated hnRNPA2B1 controls the sorting of miRNAs into exosomes through binding to specific motifs. Nat. Commun..

[36] Garcia-Martin R., Wang G., Brandão B.B., Zanotto T.M., Shah S., Kumar Patel S., Schilling B., Kahn C.R. (2022). MicroRNA sequence codes for small extracellular vesicle release and cellular retention. Nature.

[37] Mehterov N., Sacconi A., Pulito C., Vladimirov B., Haralanov G., Pazardjikliev D., Nonchev B., Berindan-Neagoe I., Blandino G., Sarafian V. (2022). A novel panel of clinically relevant miRNAs signature accurately differentiates oral cancer from normal mucosa. Front. Oncol..

[38] Panvongsa W., Pegtel D.M., Voortman J. (2022). More than a Bubble: Extracellular Vesicle microRNAs in Head and Neck Squamous Cell Carcinoma. Cancers.

[39] Rybarczyk A., Lehmann T., Iwańczyk-Skalska E., Juzwa W., Pławski A., Kopciuch K., Blazewicz J., Jagodziński P.P. (2023). In silico and in vitro analysis of the impact of single substitutions within EXO-motifs on Hsa-MiR-1246 intercellular transfer in breast cancer cell. J. Appl. Genet..

[40] Ma L., Singh J., Schekman R. (2023). Two RNA-binding proteins mediate the sorting of miR223 from mitochondria into exosomes. eLife.

[41] Shurtleff M.J., Temoche-Diaz M.M., Karfilis K.V., Ri S., Schekman R. (2016). Y-box protein 1 is required to sort microRNAs into exosomes in cells and in a cell-free reaction. eLife.

[42] Xu R., Yu Z.-L., Liu X.-C., Xie Q.-H., Wu M., Chen G. (2023). Aptamer-Assisted Traceless Isolation of PD-L1-Positive Small Extracellular Vesicles for Dissecting Their Subpopulation Signature and Function. Anal. Chem..

[43] Satake T., Suetsugu A., Nakamura M., Kunisada T., Saji S., Moriwaki H., Shimizu M., Hoffman R.M. (2019). Color-coded Imaging of the Fate of Cancer-cell-derived Exosomes During Pancreatic Cancer Metastases in a Nude-mouse Model. Anticancer Res..

[44] Rodríguez M., Silva J., López-Alfonso A., López-Muñiz M.B., Peña C., Domínguez G., García J.M., López-Gónzalez A., Méndez M., Provencio M. (2014). Different exosome cargo from plasma/bronchoalveolar lavage in non-small-cell lung cancer. Genes Chromosomes Cancer.

[45] Rayner K.J., Hennessy E.J. (2013). Extracellular communication via microRNA: Lipid particles have a new message. J. Lipid Res..

[46] Leary N., Walser S., He Y., Cousin N., Pereira P., Gallo A., Collado-Diaz V., Halin C., Garcia-Silva S., Peinado H. (2022). Melanoma-derived extracellular vesicles mediate lymphatic remodelling and impair tumour immunity in draining lymph nodes. J. Extracell. Vesicles.

[47] Su X., Brassard A., Bartolomucci A., Dhoparee-Doomah I., Qiu Q., Tsering T., Rohanizadeh R., Koufos O., Giannias B., Bourdeau F. (2023). Tumour extracellular vesicles induce neutrophil extracellular traps to promote lymph node metastasis. J. Extracell. Vesicles.

[48] Setlai B.P., Hull R., Reis R.M., Agbor C., Ambele M.A., Mulaudzi T.V., Dlamini Z. (2022). MicroRNA Interrelated Epithelial Mesenchymal Transition (EMT) in Glioblastoma. Genes.

[49] Hsu Y.L., Hung J.Y., Chang W.A., Lin Y.S., Pan Y.C., Tsai P.H., Wu C.Y., Kuo P.L. (2017). Hypoxic lung cancer-secreted exosomal miR-23a increased angiogenesis and vascular permeability by targeting prolyl hydroxylase and tight junction protein ZO-1. Oncogene.

[50] Giusti I., Poppa G., Di Fazio G., D’Ascenzo S., Dolo V. (2023). Metastatic Dissemination: Role of Tumor-Derived Extracellular Vesicles and Their Use as Clinical Biomarkers. Int. J. Mol. Sci..

[51] Reed T., Schorey J., D’Souza-Schorey C. (2021). Tumor-Derived Extracellular Vesicles: A Means of Co-opting Macrophage Polarization in the Tumor Microenvironment. Front. Cell Dev. Biol..

[52] Soriani A., Vulpis E., Cuollo L., Santoni A., Zingoni A. (2020). Cancer extracellular vesicles as novel regulators of NK cell response. Cytokine Growth Factor Rev..

[53] Fernández-Messina L., Gutiérrez-Vázquez C., Rivas-García E., Sánchez-Madrid F., de la Fuente H. (2015). Immunomodulatory role of microRNAs transferred by extracellular vesicles. Biol. Cell.

[54] Verro B., Saraniti C., Carlisi D., Chiesa-Estomba C., Maniaci A., Lechien J.R., Mayo M., Fakhry N., Lauricella M. (2023). Biomarkers in Laryngeal Squamous Cell Carcinoma: The Literature Review. Cancers.

