# Supplementary material for: A Group of Highly Secretory miRNAs Correlates with Lymph Node Metastasis and Poor Prognosis in Oral Squamous Cell Carcinoma
Source: Biomolecules. 2024 Feb 15;14(2):224. doi: 10.3390/biom14020224 (PMC10886572; doi:10.3390/biom14020224)
Supplement: Supplementary file 1 [file biomolecules-14-00224-s001.zip › Supplementary Materials Figures.pdf]

Supplementary Figures

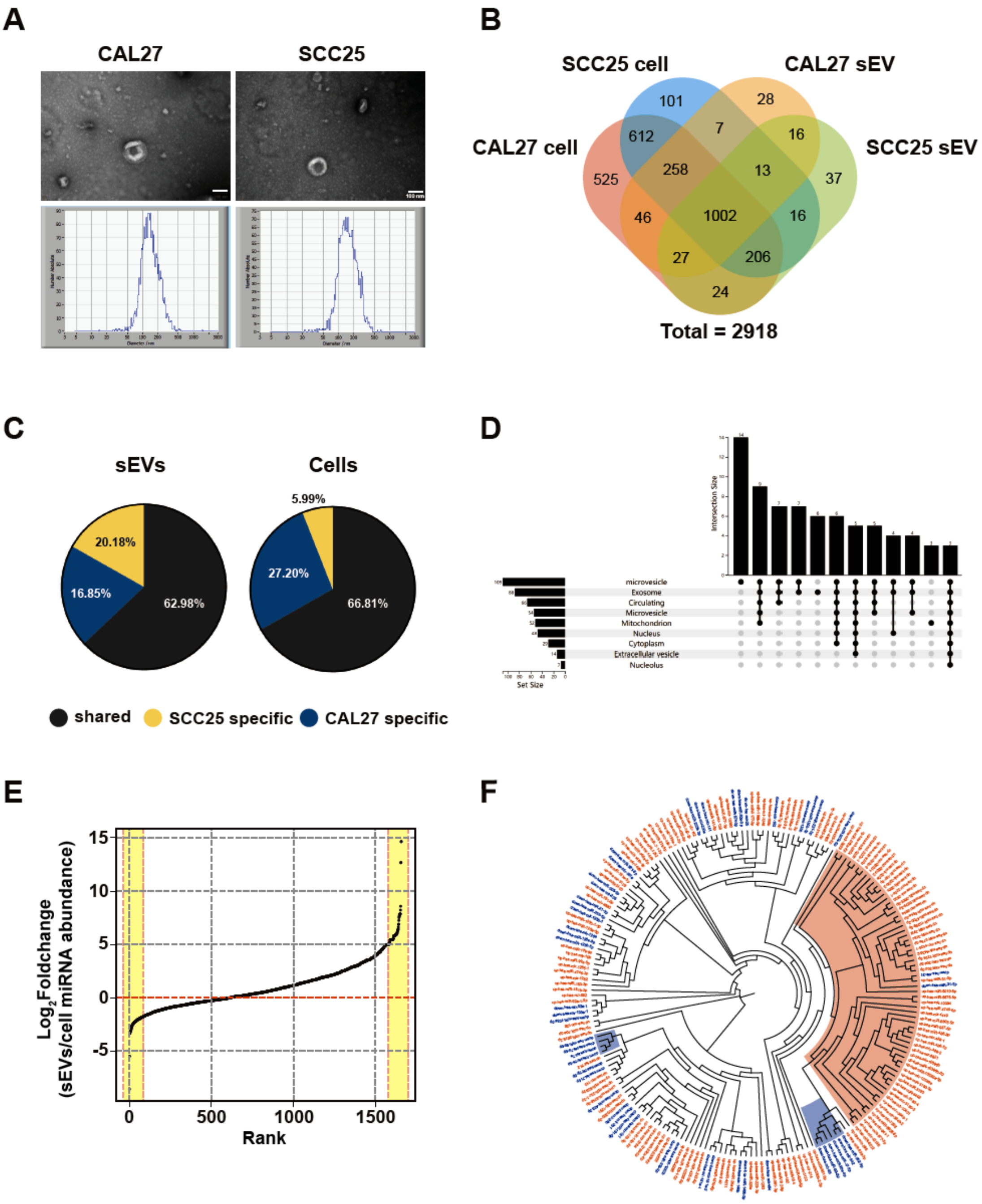

Figure S1. Analysis of miRNA profiles in OSCC cells and sEVs. (**A**) TEM images and NTA analysis of sEVs extracted from CAL27 and SCC25. Scale bar represents 100 nanometers. (**B**) Venn diagram showing the overlap and specificity of miRNAs between CAL27 cells, CAL27-derived sEVs, SCC25 cells, and SCC25-derived sEVs. The total number of identified miRNAs is shown. (**C**) Pie charts comparing the percentage of shared and cell line-specific miRNAs in cells and sEVs. The left pie chart represents the distribution of miRNAs in sEVs. The right pie chart represents the distribution of miRNAs in cells. (**D**) UpSet plot shows the RNA location enrichment analysis of C1 and C2 group miRNAs. (**E**) Ranked plot showing the fold-change of miRNA abundance in sEVs versus cells. The x-axis represents the rank of miRNAs based on differential abundance; the y-axis shows the log-transformed fold-change (sEVs vs cells). The highlighted yellow area indicates miRNAs with significant differential expression. (**F**)

Phylogenetic tree displaying the clustering of miRNAs based on their sequence homology. sEVs miRNAs were colored in red and cellular miRNAs were colored in blue. Orange block denotes sEVs miRNAs dominant clusters and blue block denotes cellular miRNA dominant clusters.

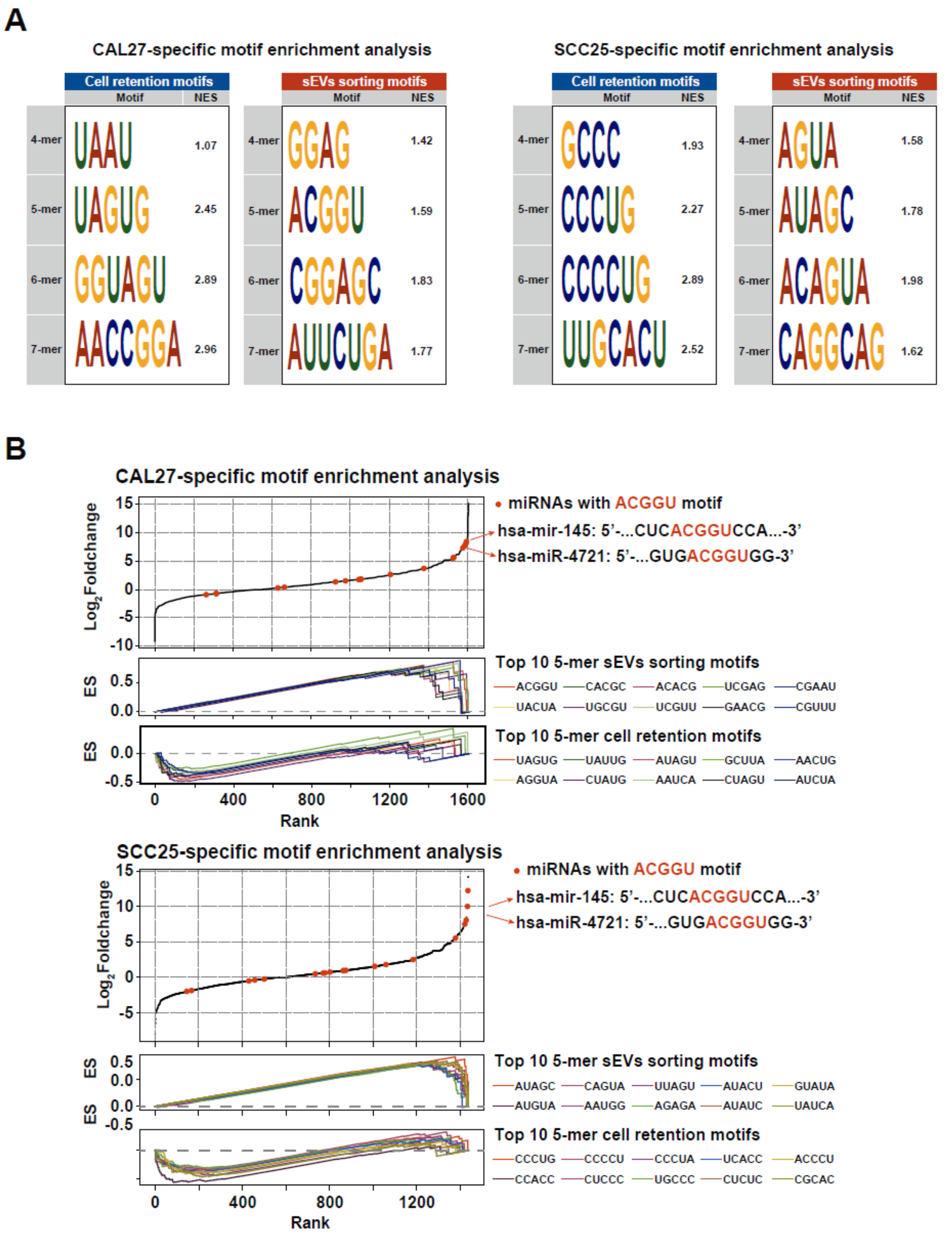

Figure S2. Motif enrichment analysis of secretory and cellular miRNAs specific to CAL27 and SCC25. (**A**) Motifs enriched in miRNAs retained in OSCC cells or sorted into sEVs, specific to CAL27 and SCC25, respectively. Normalized enrichment score (NES) is provided for each motif, indicating the degree of enrichment of each motif. (**B**) Running enrichment score plot of top 10 5-mer sEVs sorting and cell retention motifs, respectively. Red dots represent miRNAs containing a specific motif. Each colored line

illustrates one of the top motif distributions across the ranked miRNAs.

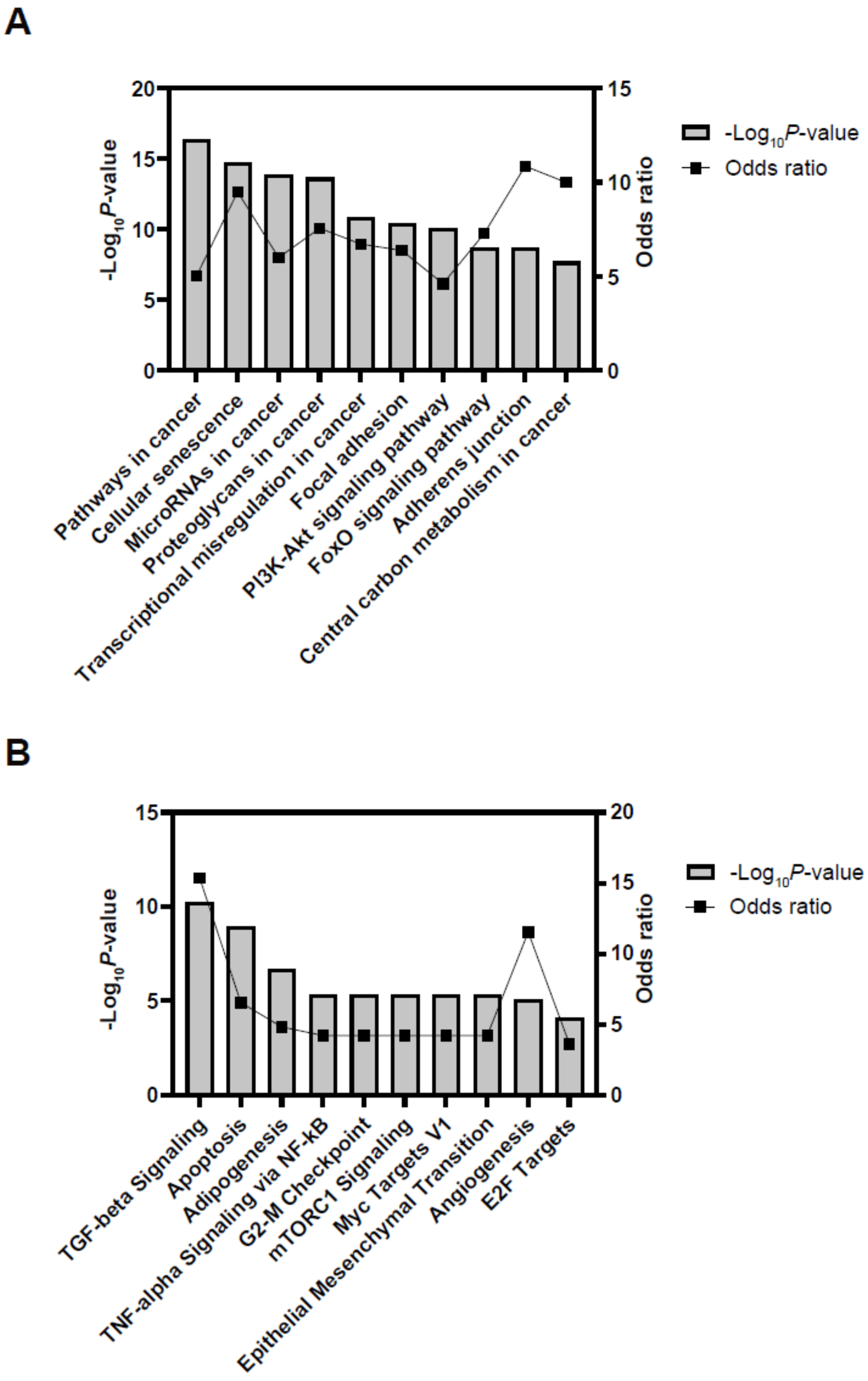

Figure S3. Functional enrichment analysis of sEVs miRNAs. (**A**) KEGG pathway enrichment analysis of sEVs miRNAs. (**B**) Molecular function enrichment (MSigDB Hallmark) analysis of sEVs miRNAs. Bars represented -log$_{10}$*P*-value, indicating the significance of each pathway's enrichment. Lines showed the corresponding odds ratio for each pathway.

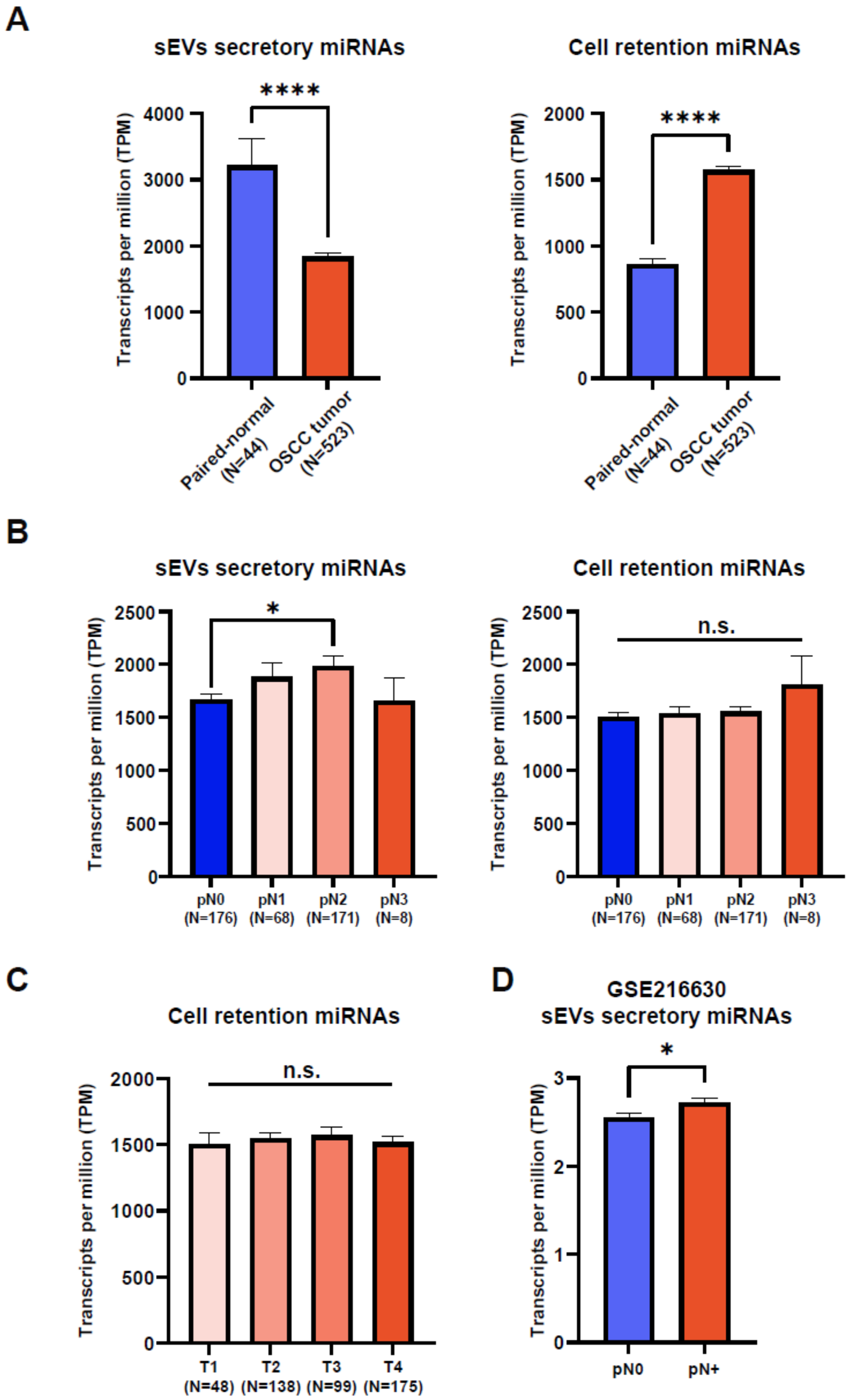

Figure S4. Clinical relevance of sEVs secretory and cellular retention miRNAs in TCGA-HNSC dataset. (**A**) Expression levels of sEVs secretory and cell retention miRNAs in normal and tumor tissues. **** *P*-value < 0.0001. (**B**) Expression levels of sEVs secretory and cell retention miRNAs in patients with different

N stages (N0-N3). (**C**) Expression levels of sEVs secretory and cell retention miRNAs in patients with different T stages (T1-T3). (**D**) Expression levels of sEVs secretory and cell retention miRNAs in patients with different N stages (N0 or N+) in GSE216630 dataset. * *P*-value < 0.05. n.s. not significant.
